# Imagining a Supportive Close Person and Sustained Engagement in a Cognitive Task: A Repeated-Measures Study

**DOI:** 10.3390/brainsci16101034

**Published:** 2026-09-27

**Authors:** Wojciech Styk, Ewa Wojtowicz, Ewa Humeniuk

**Affiliations:** 1 Academic Laboratory of Psychological Tests, Medical University of Lublin, 20-059 Lublin, Poland; 2 Department of Pedeutology and Educational Psychology, Christian Theological Academy in Warsaw, 01-771 Warsaw, Poland

**Keywords:** close relationships, mental imagery, social support, sustained task engagement, persistence, repeated-measures design, the maze test

## Abstract

Background: Close relationships may support goal-directed behavior not only through direct interpersonal contact but also through the activation of mental representations of supportive others. However, relatively little is known about whether imagining a supportive close person is associated with sustained behavioral engagement during a cognitive task. Objective: This study examined whether supportive-close-person imagery is associated with higher behavioral indicators of sustained task engagement compared with a non-social control condition and a socially neutral familiar-person imagery condition. Methods: Eighty adults participated in a within-subject repeated-measures study. Each participant completed The Maze Test in three counterbalanced conditions: observation of a neutral object, imagery of a socially neutral familiar person, and imagery of a supportive close person. The primary behavioral indicators were total task time and the number of completed mazes. Average time per maze was treated as an auxiliary indicator of task pace or efficiency. The DASS-42 was administered after each session as a symptom-stability screening measure. Results: Experimental condition had a significant effect on all behavioral outcomes. Participants showed the longest total task time and completed the highest number of mazes in the supportive-close-person condition, intermediate values in the socially neutral familiar-person condition, and the lowest values in the control condition. DASS-42 scores, session number, and condition order did not account for the observed pattern. A censoring-aware Cox model also confirmed the robustness of the condition effect on total task time despite the automatic 1500 s task limit. Conclusions: Imagining a supportive close person was associated with greater sustained engagement in a cognitive task. The findings are consistent with the possibility that close-relationship representations may support goal-directed behavior, although the psychological mechanisms underlying this effect require direct examination in future studies.

## 1. Introduction

Persistence in action is commonly understood as the ability to continue an activity despite obstacles, failure, fatigue, or a lack of immediate reward. It constitutes an important psychological resource supporting the pursuit of long-term goals [1,2]. In psychological literature, persistence is closely related to constructs such as self-control, self-regulation, mental resilience, and grit, the latter combining passion with perseverance toward long-term goals [3,4,5]. Recent theoretical approaches further distinguish between two forms of persistence: flexible persistence and rigid persistence. Flexible persistence, rooted in harmonious passion, involves striving toward a goal while maintaining openness and the ability to engage in other life activities. In this context, harmonious passion refers to a flexible and autonomous form of involvement in an activity that remains integrated with other areas of life. In contrast, rigid persistence, driven by obsessive passion, is associated with a narrow and controlling focus on a specific task and may lead to neglect of other goals and life domains [6].

Research indicates that higher levels of persistence are associated with better academic outcomes [5,7,8], better mental health [9], and greater life satisfaction [2,8]. Persistence is not only a stable individual tendency but also a dynamic process shaped by situational, cognitive, emotional, and interpersonal factors. Sustained goal-directed behavior requires self-regulatory processes, including the ability to maintain effort, regulate attention, and manage emotions that may emerge in the face of difficulty [10]. Thus, persistence can be strengthened or weakened depending on the psychological context in which action takes place.

One important contextual factor that may support persistence is social support. Social support may take instrumental, emotional, informational, or appraisal-related forms [11]. It refers both to objectively available assistance and to the belief that one can rely on others when needed [12]. Perceived social support may reduce the subjective cost of effort, facilitate emotion regulation, and support continued engagement in demanding activities [13,14,15]. Broad meta-analytic evidence indicates that perceived social support is positively associated with mental and physical health as well as educational and occupational functioning [16]. In close relationships, responsive support and practical partner assistance are also associated with stronger goal engagement and greater progress toward personal goals [17].

Support does not have to operate only through direct interpersonal contact. Close relationships may also influence behavior through internalized mental representations of significant others. Psychophysiological and neuroimaging studies suggest that contact with a close person or activation of their mental representation may support emotion regulation and reduce stress reactivity. These processes have been linked to systems involved in safety, reward processing, and stress-response regulation [18,19]. From this perspective, close-relationship representations may function as internal regulatory resources that can be activated even when the close person is physically absent.

This assumption is consistent with research on mental simulation. Mental simulation refers to imagining specific situations, actions, outcomes, or people in a way that can influence emotions, motivation, and behavior [20,21,22]. Studies show that imagining or being reminded of a close and supportive person can produce psychological and physiological effects similar to those observed during actual interpersonal contact. For example, viewing a partner’s photograph, writing about a close person, or holding an object associated with a relationship may elicit emotional and physiological responses linked to safety, comfort, or reduced distress [18,23,24,25]. Viewing a photograph of a close person has also been shown to reduce experimentally induced physical pain [26], and brief activation of partner representations may buffer affective responses to everyday stressors [27].

The activation of close-person representations may also influence goal-directed behavior. Fitzsimons and Bargh [28] showed that thinking about a close relationship partner can activate interpersonal goals associated with that person and influence persistence in difficult tasks. Similarly, attachment-security priming has been associated with improved affective functioning and reduced negative emotional states [29], whereas repeated security priming in daily life has been shown to reduce perceived stress and strengthen resilience-related outcomes [30]. Mikulincer et al. [31] also demonstrated that activating a sense of attachment security can support creative problem solving. Together, these findings suggest that close-relationship representations may be relevant not only for emotional regulation but also for self-regulation and sustained task engagement.

However, only a limited number of studies have directly examined whether mental simulation involving another person affects behavioral persistence during a standardized task. Styk et al. [32] showed that mental simulation may support persistence in the context of health-related behavior among individuals with different BMI levels. Pei et al. [33] also demonstrated that brief activation of support-related representations can influence emotional responses. Nevertheless, relatively little is known about whether imagining a supportive close person is associated with behavioral indicators of sustained engagement in a cognitive task. It also remains unclear whether any such effect is specific to the activation of a supportive close relationship or whether it may partly reflect a broader effect of activating a social representation.

The distinction between a supportive close person and a socially neutral familiar person is therefore theoretically important. Imagining a socially neutral familiar person may activate a general social context without strong emotional closeness or explicit support. Such activation may increase attention, alertness, or social monitoring, but it does not involve the same relational meanings as imagining a supportive close person. In contrast, imagining a supportive close person may additionally activate autobiographical memory, perceived support, reassurance, emotional closeness, or self-confidence. Therefore, comparing these two imagery conditions with a non-social control condition allows for a more nuanced examination of whether sustained task engagement is associated only with close supportive imagery or also with the broader activation of another person.

At the same time, it is important to emphasize that the present study did not include a direct manipulation check. Therefore, the study cannot determine whether the observed effects, if present, are driven specifically by perceived support, imagery vividness, attachment security, positive affect, arousal, self-efficacy, or other processes. The present study should therefore be understood as testing behavioral differences between experimental conditions rather than as directly testing the psychological mechanisms underlying those differences.

### Objective

The aim of the present study was to examine whether imagining a supportive close person is associated with higher behavioral indicators of sustained task engagement during a computerized cognitive task. The study compared three experimental conditions: a non-social control condition involving observation of a neutral object, a socially neutral familiar-person imagery condition, and a supportive-close-person imagery condition. Depressive, anxiety, and stress symptoms were monitored across weekly sessions using the DASS-42 as a symptom-stability screening measure.

It was expected that supportive-close-person imagery would be associated with longer total task time and a higher number of completed mazes than both the non-social control condition and the socially neutral familiar-person imagery condition. It was also expected that the socially neutral familiar-person condition would produce intermediate values on these outcomes, higher than the non-social control condition but lower than the supportive-close-person condition. Average time per maze was treated as an auxiliary indicator of task pace or efficiency and was analyzed exploratorily.

## 2. Materials and Methods

### 2.1. Study Design and Participants

The study used a within-subject repeated-measures design. Each participant completed the same computerized cognitive task in three experimental conditions: a control condition, a socially neutral familiar-person imagery condition, and a supportive-close-person imagery condition. The conditions were completed in three separate sessions conducted at one-week intervals. The order of conditions was counterbalanced across participants.

The study included 80 adults, including 56 women and 24 men. The mean age of the participants was 29.4 years (SD = 9.8; range: 18–56 years). Participants were recruited primarily among students and university employees using convenience sampling. Recruitment and data collection took place during the winter semester, between October and December 2025. The study was conducted in a university computer room using a standardized computerized task. Participants did not receive financial compensation for participation.

The target sample size was determined by feasibility and by the planned recruitment period during the winter semester. The aim was to recruit approximately 80 participants, which allowed broadly balanced allocation across the six possible counterbalanced condition sequences. No a priori power analysis was conducted.

Participants were eligible for the study if they were adults, were able to independently complete a computerized cognitive task, and provided informed consent to participate. Exclusion criteria were failure to complete all three experimental sessions, incomplete data from The Maze Test or the DASS-42, or a substantial increase in depressive, anxiety, or stress symptoms between sessions, defined as a shift into the elevated, high, or very high range according to Polish DASS-42 sten norms.

In the analyzed dataset, no participant shifted into the elevated, high, or very high range on the DASS-42; therefore, the full sample of 80 participants was included in the statistical analyses. Participants were informed about the aim and course of the study, the voluntary nature of participation, and their right to withdraw from the study at any stage without giving a reason.

### 2.2. Measures

#### 2.2.1. The Maze Test

The Maze Test is a computerized research tool designed to assess persistence understood as sustained engagement in a task despite monotony, effort, or difficulty [34]. In the task, participants solve simple maze problems using a computer mouse. Each maze begins at a starting point, and the participant is asked to find the path to the exit while avoiding incorrect routes.

The task consisted of a pool of 200 maze stimuli presented in randomized order. If the number of completed mazes exceeded the number of available unique stimuli, mazes could be repeated. The task had an automatic maximum duration of 1500 s, corresponding to 25 min. Participants could also terminate the task earlier at any point. Thus, total task time reflected voluntary sustained engagement within the fixed time limit of the tool.

The following behavioral indicators were calculated: (1) number of completed mazes, defined as the number of successfully completed maze tasks and reflecting both continued task engagement and task performance; (2) total task time, defined as the total time spent on the task in seconds and treated as a primary indicator of sustained task engagement because participants could stop the task before reaching the maximum duration; and (3) average time per maze, defined as total task time divided by the number of completed mazes and treated as an auxiliary indicator of task pace or efficiency rather than as an independent indicator of persistence, because it is mathematically derived from the two previous variables [35].

These indicators should not be interpreted as pure measures of persistence. Although they capture sustained behavioral engagement in the task, they may also reflect processing speed, visuospatial ability, cognitive strategy, task familiarity, and general cognitive performance.

#### 2.2.2. DASS-42

The Depression Anxiety Stress Scales-42 (DASS-42) was used as a symptom-stability screening measure. The questionnaire includes 42 items assessing three negative emotional states: depression, anxiety, and stress. Each scale contains 14 items. The Polish version of the DASS-42 has been adapted and evaluated for validity and reliability, and Polish sten norms are available [36].

In the present study, the DASS-42 was not used as a manipulation check, a pre-post measure, or an assessment of momentary affective response to the experimental conditions. The standard DASS-42 instruction referring to symptoms experienced during the previous week was retained. Therefore, the purpose of the questionnaire was to determine whether participants entered subsequent weekly sessions with substantially changed depressive, anxiety, or stress symptoms. To avoid directing participants’ attention toward negative affective states before the experimental manipulation and Maze Test performance, the DASS-42 was administered after completion of The Maze Test in each session.

### 2.3. Experimental Procedure

Each participant completed three experimental sessions separated by one-week intervals. In each session, participants first received one of the experimental instructions, then completed The Maze Test, and finally completed the DASS-42. The same general procedure was used in all sessions.

The order of the three experimental conditions was counterbalanced across participants using the six possible condition sequences: ABC, ACB, BAC, BCA, CAB, and CBA. Participants were allocated to sequences in a broadly balanced manner, with 13 or 14 participants assigned to each sequence. This procedure was used to reduce the risk that the observed effects would be attributable to practice, fatigue, habituation, carryover effects, or the order of condition presentation.

The imagery and observation procedures were based on standardized written instructions. The procedures were self-paced and were not strictly time-limited. Participants proceeded through the steps of the instruction at their own reading pace. The structure of the instructions was matched across conditions and consisted of four steps: initial focus, elaboration of the imagined or observed content, noticing impressions or emotional reactions, and returning attention to the present moment. Participants were free to keep their eyes open or closed during the imagery procedures.

No direct manipulation check was included. Therefore, the study did not directly assess imagery vividness, perceived support, emotional closeness, affective valence, arousal, self-efficacy, or attachment security after the experimental instructions.

#### 2.3.1. Control Condition: Observation of a Neutral Object

In the control condition, participants were asked to choose and observe a neutral object in their surroundings, such as a pen, cup, book, or clock. They were instructed to focus on the object’s shape, color, texture, and visible details, and then to count or identify as many features of the object as possible. This condition was designed as a non-social, emotionally neutral comparison condition.

#### 2.3.2. Socially Neutral Familiar-Person Imagery Condition

In the socially neutral familiar-person condition, participants were asked to imagine a person known from everyday life but not associated with a close emotional bond. Examples included a neighbor, shop assistant, bus driver, or another person encountered in everyday situations. Participants were instructed to imagine the person’s appearance, clothing, facial expression, possible movements, and neutral everyday context. This condition was designed to activate a social but non-close and relatively emotionally neutral representation.

#### 2.3.3. Supportive-Close-Person Imagery Condition

In the supportive-close-person condition, participants were asked to imagine a close person who was a source of support for them. This could be a family member, friend, partner, or another person associated with support and safety. Participants were instructed to imagine the person’s appearance, facial expression, tone of voice, gestures, and a situation in which this person provided help or reassurance.

This condition included more explicitly emotional and supportive content than the other two conditions, including imagined supportive words, reassurance, and possible physical closeness, such as a hug or holding hands. Therefore, the condition differed from the other conditions not only in the type of imagined person but also in emotional valence, autobiographical recall, reassurance, and potential self-efficacy cues. This feature was considered in the interpretation of the findings and is addressed as a limitation of the study.

The overall study procedure in the within-subject repeated-measures design is shown in Figure 1.

### 2.4. Statistical Analysis

The primary aim of the statistical analysis was to examine whether behavioral indicators of sustained task engagement differed across the three experimental conditions: control condition, socially neutral familiar-person imagery condition, and supportive-close-person imagery condition. Because each participant completed all three conditions, repeated-measures analyses of variance (ANOVAs) were conducted separately for the behavioral outcomes.

The primary indicators of sustained task engagement were total task time and the number of completed mazes. Average time per maze was treated as an auxiliary indicator of task pace or efficiency, because it was mathematically derived from total task time and the number of completed mazes. Therefore, this variable was interpreted more cautiously and was not treated as an independent indicator of persistence.

Sex was not included in the main analytical model. Although sex had initially been examined as a possible exploratory control variable, no sex-related hypothesis was formulated, and the sample was unbalanced with respect to sex. Therefore, the final primary analyses focused on the within-subject effect of experimental condition.

For each repeated-measures ANOVA, the assumption of sphericity was evaluated using Mauchly’s test. When the sphericity assumption was violated, Greenhouse-Geisser-corrected degrees of freedom and *p* values were reported. Pairwise comparisons between conditions were performed using paired-samples t tests with Bonferroni correction for multiple comparisons. For pairwise comparisons, effect sizes were reported as Cohen’s dav, calculated by dividing the mean paired difference by the average standard deviation of the two compared conditions. This effect-size index was selected as a more conservative standardized estimate than dz, because effect sizes based on the standard deviation of paired differences may become inflated when within-person changes are highly consistent.

The DASS-42 was used as a symptom-stability screening measure, not as a manipulation check or a measure of momentary affective response to the experimental conditions. Because the standard DASS-42 instruction refers to symptoms experienced during the previous week, DASS-42 scores were used to evaluate whether participants entered subsequent sessions with substantially changed depressive, anxiety, or stress symptoms. To avoid directing participants’ attention toward negative affective states before the experimental manipulation and task performance, the DASS-42 was administered after The Maze Test. Continuous DASS-42 scores were also compared across experimental conditions using repeated-measures ANOVAs.

Because total task time was right-censored at 1500 s, an additional censoring-aware sensitivity analysis was conducted for this outcome. A Cox proportional hazards model was used, with voluntary task termination before 1500 s treated as the event and reaching the 1500 s limit treated as right-censoring. Experimental condition was entered as the predictor. Because each participant contributed observations in all three conditions, cluster-robust standard errors were calculated using participant ID as the clustering variable. In this model, hazard ratios below 1 indicate a lower likelihood of terminating the task at a given time point, and therefore greater sustained engagement.

Additional sensitivity analyses were conducted to evaluate whether the main condition effects could be explained by session number or by the order of condition presentation. These analyses were used to assess possible practice, habituation, fatigue, or carryover effects associated with repeated completion of The Maze Test.

The significance level was set at α = 0.05. Analyses were conducted in R, version 4.4.2.

## 3. Results

### 3.1. Symptom Stability Based on DASS-42 Scores

The DASS-42 was administered after each session using the standard instruction referring to the previous week. Raw scores were converted into sten categories according to the Polish adaptation and preliminary normalization of the instrument. A potentially relevant symptom change was defined as a participant’s transition into the elevated, high, or very high range, corresponding to stens 7–10. No such transition was observed in any participant. The maximum overall sten score was 6; therefore, all participants remained within the low or average range and were included in the analyses.

Continuous DASS-42 scores were also compared across experimental conditions. No significant condition effect was observed for depression, F(2, 158) = 2.22, *p* = 0.112, η^2^p = 0.027; anxiety, F(2, 158) = 0.43, *p* = 0.651, η^2^p = 0.005; stress, F(2, 158) = 0.96, *p* = 0.387, η^2^p = 0.012; total DASS-42 score, F(2, 158) = 1.92, *p* = 0.150, η^2^p = 0.024; or overall sten score, F(2, 158) = 1.00, *p* = 0.370, η^2^p = 0.012. These results indicate that depressive, anxiety, and stress symptoms did not differ significantly between experimental conditions.

### 3.2. Descriptive Statistics and Ceiling Effects

Descriptive statistics are presented in Table 1. The number of completed mazes and total task time were lowest in the control condition, higher in the socially neutral familiar-person imagery condition, and highest in the supportive-close-person imagery condition. Average time per maze was similar in the control and socially neutral familiar-person conditions and lower in the supportive-close-person condition.

The Maze Test had an automatic maximum duration of 1500 s, corresponding to 25 min. Participants could terminate the task earlier at any point. The 1500 s ceiling was reached by 2 participants in the control condition (2.5%), 14 participants in the socially neutral familiar-person condition (17.5%), and 40 participants in the supportive-close-person condition (50.0%). Therefore, total task time should be interpreted as sustained task engagement within the time limit of the tool. The presence of this upper limit was taken into account in the interpretation of the results.

### 3.3. Main Repeated-Measures ANOVAs

Repeated-measures ANOVAs showed significant effects of experimental condition for all three behavioral outcomes.

For the number of completed mazes, Mauchly’s test did not indicate a violation of sphericity, W = 0.944, *p* = 0.109. The effect of condition was significant, F(2, 158) = 996.99, *p* < 0.001, η^2^p = 0.927. Participants completed the fewest mazes in the control condition, more mazes in the socially neutral familiar-person condition, and the most mazes in the supportive-close-person condition.

For total task time, Mauchly’s test indicated a violation of sphericity, W = 0.903, *p* = 0.020; therefore, Greenhouse-Geisser-corrected results are reported. The effect of condition was significant, F(1.82, 144.07) = 208.71, *p* < 0.001, η^2^p = 0.725. Participants spent the shortest time on the task in the control condition, longer in the socially neutral familiar-person condition, and longest in the supportive-close-person condition. Because the task duration was capped at 1500 s, this result should be interpreted as a difference in sustained engagement within the fixed time limit of the task.

For average time per maze, Mauchly’s test also indicated a violation of sphericity, W = 0.736, *p* < 0.001; therefore, Greenhouse-Geisser-corrected results are reported. The effect of condition was significant, F(1.58, 124.98) = 3.81, *p* = 0.034, η^2^p = 0.046. Average time per maze was lower in the supportive-close-person condition than in the two remaining conditions. The complete repeated-measures ANOVA results, including degrees of freedom, test statistics, p values, effect sizes, and sphericity corrections, are presented in Table 2.

### 3.4. Pairwise Comparisons

Bonferroni-adjusted pairwise comparisons showed that all three conditions differed significantly from one another for the number of completed mazes and total task time. For both outcomes, the lowest values were observed in the control condition, intermediate values in the socially neutral familiar-person condition, and the highest values in the supportive-close-person condition.

For average time per maze, there was no significant difference between the control and socially neutral familiar-person conditions. After Bonferroni correction, the supportive-close-person condition did not differ significantly from the control condition, but it was associated with a lower average time per maze than the socially neutral familiar-person condition. Pairwise comparisons between experimental conditions are presented in Table 3.

### 3.5. Sensitivity Analyses: Session and Order Effects

Additional sensitivity analyses were conducted to examine whether the observed effects could be attributed to session number or to the order of condition presentation. The six possible counterbalanced condition sequences were represented in the sample: ABC, ACB, BAC, BCA, CAB, and CBA. The number of participants assigned to the sequences was broadly balanced, with 13 or 14 participants per sequence.

Adding session number to the models did not significantly improve model fit for the number of completed mazes, *p* = 0.954; total task time, *p* = 0.0699; or average time per maze, *p* = 0.766. Thus, the results did not indicate a general practice, habituation, or fatigue effect across the three sessions.

The effect of condition order was also examined. No significant order effect was observed for the number of completed mazes, *p* = 0.732; total task time, *p* = 0.300; or average time per maze, *p* = 0.685. In addition, condition × order interactions were not significant for the number of completed mazes, *p* = 0.799; total task time, *p* = 0.276; or average time per maze, *p* = 0.700. These results suggest that the main condition effects were not attributable to the sequence in which the experimental conditions were completed.

### 3.6. Censoring-Aware Sensitivity Analysis for Total Task Time

To examine whether the condition effect on total task time was robust to right-censoring at 1500 s, a Cox proportional hazards sensitivity analysis was conducted. The model showed a significant overall effect of condition, robust Wald χ^2^(2) = 115.73, *p* < 0.001. Compared with the control condition, the likelihood of voluntary task termination was lower in the socially neutral familiar-person condition, HR = 0.38, 95% CI [0.29, 0.49], *p* < 0.001, and substantially lower in the supportive-close-person condition, HR = 0.15, 95% CI [0.11, 0.21], *p* < 0.001. The supportive-close-person condition was also associated with a lower likelihood of task termination than the socially neutral familiar-person condition, HR = 0.39, 95% CI [0.31, 0.50], *p* < 0.001. These results indicate that the condition effect on total task duration remained robust when the right-censored nature of the outcome was taken into account.

### 3.7. Summary of Results

Overall, the findings showed a consistent effect of experimental condition on behavioral indicators of sustained task engagement. The supportive-close-person imagery condition was associated with the highest number of completed mazes and the longest total task time, whereas the control condition was associated with the lowest values. The socially neutral familiar-person condition generally produced intermediate results.

The additional analyses did not indicate that the main findings were explained by differences in DASS-42 symptom levels, session number, or condition order. In addition, the censoring-aware Cox proportional hazards analysis confirmed that the condition effect on total task time remained robust when reaching the 1500 s limit was treated as right-censoring. Average time per maze should also be interpreted cautiously, because it is mathematically derived from total time and number of completed mazes and reflects task pace or efficiency rather than persistence alone.

## 4. Discussion

The aim of the present study was to examine whether imagining a supportive close person is associated with behavioral indicators of sustained task engagement compared with imagining a socially neutral familiar person and a non-social control condition. The results supported the main hypothesis. Participants completed the highest number of mazes and spent the longest time on the task in the supportive-close-person condition. Intermediate values were observed in the socially neutral familiar-person condition, whereas the lowest values were obtained in the control condition. This pattern suggests that the mental activation of a supportive close relationship may be associated with greater sustained engagement in a cognitive task.

These findings are consistent with research emphasizing perceived social support as an important factor related to persistence, goal pursuit, and self-regulation. Social support may reduce the subjective cost of effort, facilitate emotion regulation, and support continued engagement in demanding activities [12,13,15]. Previous studies have also shown that partner support and perceived support are associated with goal progress and everyday self-regulatory processes [17,37]. The present findings are broadly compatible with this literature, but they should be interpreted cautiously. The study did not measure perceived support directly after the manipulation; therefore, it cannot establish that perceived support itself was the mechanism responsible for the observed behavioral differences.

The finding that the socially neutral familiar-person condition produced higher values than the non-social control condition is also theoretically interesting. It suggests that the activation of a social representation, even one not associated with emotional closeness, may increase task engagement relative to focusing on a neutral object. This result is compatible with the idea that mental representations of other people can influence behavior even in the absence of direct interpersonal contact [28]. However, the present study cannot determine whether the effect of the socially neutral familiar-person condition resulted from social attention, increased alertness, imagined observation, demand characteristics, or other processes. Therefore, this condition should be interpreted as a socially neutral comparison condition rather than as a pure control for all non-specific effects of imagery.

The stronger effect observed in the supportive-close-person condition may indicate that emotional closeness and supportive relational content add to the effect of merely activating a social representation. This interpretation is consistent with previous research showing that close-relationship representations can influence affective and physiological responses. For example, viewing a partner’s photograph has been shown to reduce experimentally induced pain [23,26], and attachment figures may activate neural systems involved in safety and threat regulation [18,19]. Attachment-security priming has also been associated with increased positive affect and reduced negative affect [29], while repeated security priming has been shown to reduce perceived stress and increase resilience-related outcomes in daily life [30]. The present study extends this line of research by focusing on behavioral task engagement, but it does not directly test the affective, relational, or neurobiological mechanisms that may explain the effect.

The results may also be considered in light of research on mental simulation and interpersonal goal activation. Mental simulation can influence motivation and behavior by making specific experiences, goals, or anticipated outcomes more cognitively accessible [21,22,24]. Fitzsimons and Bargh [28] showed that activating mental representations of close relationship partners can unconsciously activate goals associated with those partners. In the present study, imagining a supportive close person may have activated meanings related to encouragement, safety, confidence, or goal pursuit. This explanation is plausible, but it remains hypothetical because goal activation, perceived support, affect, arousal, and self-efficacy were not measured after the imagery procedure.

The results for average time per maze require separate consideration. The effect of condition was significant, but weaker than the effects observed for the number of completed mazes and total task time. Average time per maze was lower in the supportive-close-person condition than in the other two conditions. This may suggest a more economical task pace in this condition; however, this variable should be interpreted cautiously. Average time per maze is mathematically derived from total task time and the number of completed mazes, and therefore it is not statistically or conceptually independent of the primary behavioral outcomes. It should be treated as an auxiliary indicator of task pace or efficiency rather than as a separate indicator of persistence.

An important methodological issue concerns the emotional structure of the experimental instructions. The supportive-close-person condition differed from the control and socially neutral familiar-person conditions not only in the type of imagined target but also in emotional content. It included autobiographical recall, supportive statements, reassurance, and possible imagined physical closeness. Therefore, the observed differences cannot be attributed uniquely to the activation of a close-person representation. They may also reflect positive affect, emotional arousal, reassurance, autobiographical memory, self-confidence, expectancy effects, or demand characteristics. This issue is a central limitation of the present study and should be addressed in future research using more closely matched control conditions.

The lack of a direct manipulation check is another important limitation. The study did not assess imagery vividness, perceived support, emotional closeness, affective valence, arousal, self-efficacy, or attachment security after the experimental instructions. As a result, it is not possible to determine whether participants engaged with the imagery procedures in the intended way or which psychological process was most strongly activated. Future studies should include direct manipulation checks immediately after the imagery procedure and before the behavioral task. Such measures would make it possible to distinguish the effects of close-person imagery from the effects of imagery vividness, positive affect, perceived support, or arousal.

The role of the DASS-42 should also be interpreted cautiously. In the present study, the DASS-42 was used as a symptom-stability screening measure across weekly sessions rather than as a manipulation check or a measure of momentary affect. No participant shifted into elevated, high, or very high sten ranges, and continuous DASS-42 scores did not differ significantly between conditions. This suggests that the main findings were not explained by substantial weekly changes in depressive, anxiety, or stress symptoms. However, because the DASS-42 refers to symptoms experienced during the previous week and was administered after the task, it cannot rule out immediate changes in affect, arousal, motivation, or self-efficacy caused by the experimental manipulation. The Polish version of the DASS-42 has been validated and normalized [36], but its role in the present study was limited to monitoring symptom stability.

Another methodological issue concerns the automatic 1500 s time limit of The Maze Test. This limit was reached by 2 participants in the control condition, 14 participants in the socially neutral familiar-person condition, and 40 participants in the supportive-close-person condition. Thus, total task time was right-censored, particularly in the supportive-close-person condition. To address this issue, we conducted an additional Cox proportional hazards sensitivity analysis in which voluntary task termination was treated as the event and reaching the 1500 s limit was treated as right-censoring. This analysis confirmed the main pattern of results: participants were less likely to terminate the task in the supportive-close-person condition than in the control and socially neutral familiar-person conditions. Therefore, the conclusion that supportive-close-person imagery was associated with greater sustained task engagement was robust to the censored nature of total task time. Nevertheless, future studies should consider longer task durations or designs without a fixed upper time limit.

The sensitivity analyses did not indicate that the main findings were explained by session number or condition order. Adding session number to the models did not significantly improve model fit, and neither order effects nor condition × order interactions were significant. These results reduce the likelihood that the observed differences were primarily attributable to practice, fatigue, habituation, or carryover effects. Nevertheless, because participants completed the same type of task in three sessions separated by one-week intervals, the possibility of repeated-testing effects cannot be entirely excluded, especially because attention and task engagement may change over time during repeated task performance [38].

The present findings should also be interpreted in light of the operationalization of persistence. The Maze Test provides behavioral indicators of sustained task engagement, including the number of completed mazes and the total time spent on the task [32,34]. However, these indicators may also reflect processing speed, visuospatial ability, task strategy, and general performance. Therefore, the present results should not be interpreted as evidence that supportive-close-person imagery increased persistence as a broad personality trait. Rather, they show that this imagery condition was associated with greater sustained engagement in a specific computerized cognitive task.

The practical implications of the findings should therefore be formulated cautiously. The results suggest that brief supportive-close-person imagery may be a promising strategy for supporting task engagement, but the present study was limited to a single laboratory cognitive task in a sample of healthy adults. It does not demonstrate effectiveness in educational, occupational, clinical, rehabilitation, or sport settings. These domains should be treated as possible areas for future research rather than as established applications. Future studies should examine whether similar effects are observed in real-life tasks, longer interventions, emotionally demanding activities, and groups differing in attachment style, relationship quality, or clinical status.

Despite these limitations, the study contributes to research on close relationships, mental simulation, and self-regulation. The within-subject design, inclusion of both non-social and socially neutral familiar-person comparison conditions, and use of behavioral outcomes strengthen the interpretation that the type of activated mental representation mattered for task engagement. The findings suggest that mentally activating a supportive close person may be associated with greater sustained engagement in goal-directed activity, while also highlighting the need for future studies that directly measure the psychological mechanisms involved.

## 5. Conclusions

Imagining a supportive close person was associated with longer task engagement and a higher number of completed mazes in a repeated-measures cognitive task. The socially neutral familiar-person condition produced intermediate outcomes, suggesting that both social representation and supportive relational content may be relevant to sustained engagement. However, the study does not identify the specific psychological mechanism underlying this effect. Because perceived support, imagery vividness, affect, arousal, self-efficacy, and attachment security were not directly measured, the findings should be interpreted as evidence of behavioral differences between experimental conditions rather than as proof of a specific motivational or relational mechanism. Future research should use more closely matched control conditions, direct manipulation checks, and task designs that address ceiling effects and right-censoring.

## Figures and Tables

**Figure 1:**
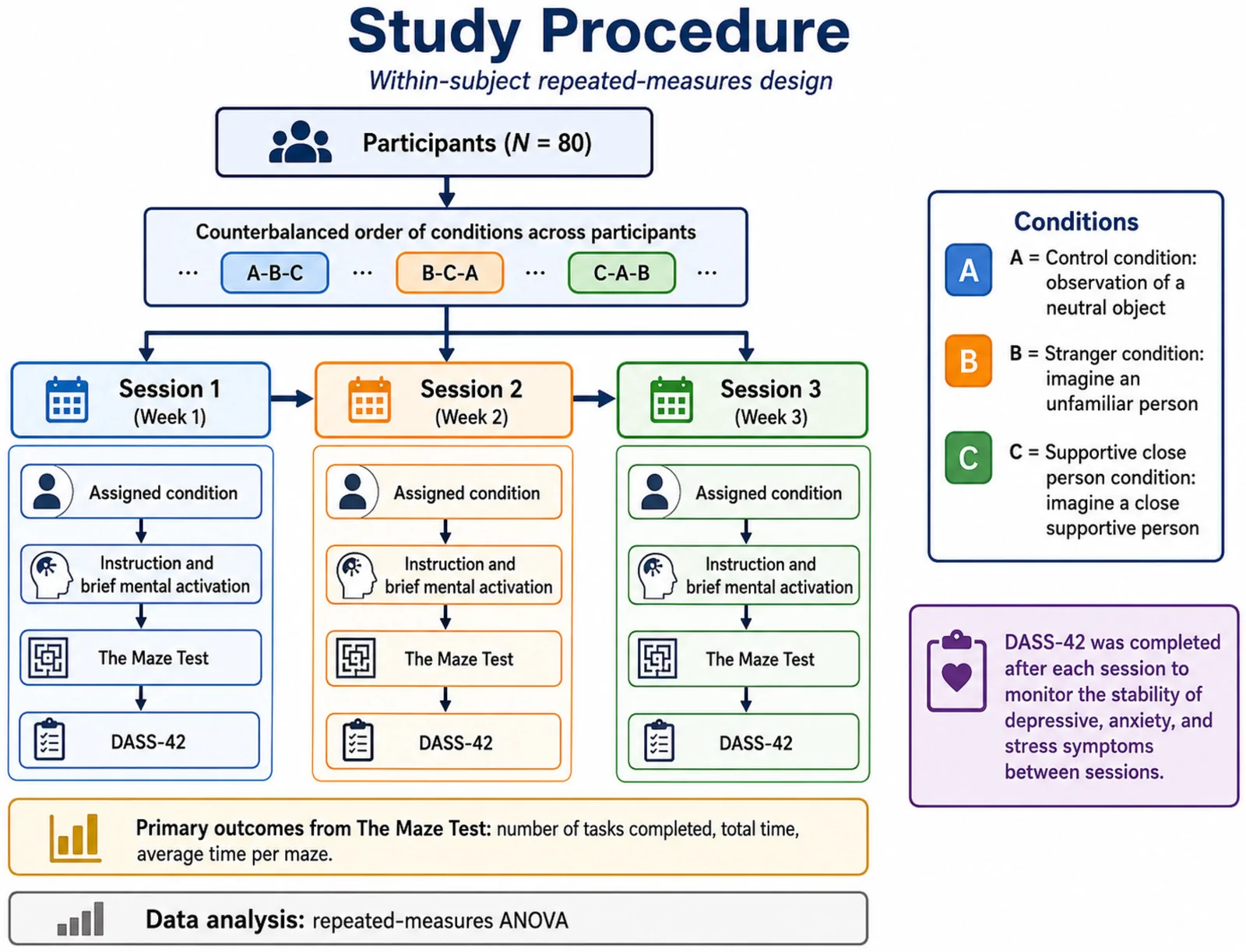
Study procedure in the within-subject repeated-measures design.

**Table 1:** Descriptive statistics and ceiling counts for behavioral outcomes across experimental conditions.

Outcome	A M (SD)	B M (SD)	C M (SD)	A Min–Max	B Min–Max	C Min–Max	Ceiling n
Completed mazes	98.35 (19.28)	122.61 (19.27)	149.21 (20.50)	64–142	60–182	87–193	-
Total task time [s]	959.40 (266.80)	1205.72 (253.30)	1368.02 (202.64)	213.60–1500.00	228.30–1500.00	577.10–1500.00	2/14/40
Average time per maze [s]	9.82 (2.42)	9.81 (1.59)	9.19 (1.00)	2.60–15.12	3.81–13.15	6.63–11.46	-

Note: A = control condition; B = socially neutral familiar-person imagery condition; C = supportive-close-person imagery condition. Ceiling counts for total task time are reported as A/B/C. The 1500 s ceiling refers only to total task time.

**Table 2:** Repeated-measures ANOVAs for behavioral outcomes.

Variable	df1	df2	F	*p*	η^2^p	Sphericity/Correction
Number of completed mazes	2.00	158.00	996.99	<0.001	0.927	Sphericity assumed
Total task time [s]	1.82	144.07	208.71	<0.001	0.725	Greenhouse-Geisser corrected
Average time per maze [s]	1.58	124.98	3.81	0.034	0.046	Greenhouse-Geisser corrected

Note: η^2^p = partial eta squared.

**Table 3:** Pairwise comparisons between experimental conditions.

Outcome	Contrast	Mean Diff.	95% CI Lower	95% CI Upper	t(79)	*p* adj.	dav
Completed mazes	B—A	24.26	21.79	26.74	19.50	<0.001	1.26
Completed mazes	C—A	50.86	48.57	53.16	44.13	<0.001	2.56
Completed mazes	C—B	26.60	24.59	28.61	26.36	<0.001	1.34
Total task time [s]	B—A	246.32	203.24	289.41	11.38	<0.001	0.95
Total task time [s]	C—A	408.63	365.53	451.73	18.87	<0.001	1.74
Total task time [s]	C—B	162.31	129.03	195.58	9.71	<0.001	0.71
Average time per maze [s]	B—A	−0.01	−0.62	0.61	−0.02	1.000	−0.00
Average time per maze [s]	C—A	−0.63	−1.18	−0.08	−2.29	0.075	−0.37
Average time per maze [s]	C—B	−0.63	−1.00	−0.25	−3.33	0.004	−0.48

Note: Mean difference was calculated as the first condition named in the contrast minus the second condition. A = control condition; B = socially neutral familiar-person imagery condition; C = supportive-close-person imagery condition. *p* adj. = *p* value adjusted using the Bonferroni correction for three comparisons within each variable. dav = Cohen’s d calculated using the average standard deviation of the two compared conditions.

## Data Availability

The anonymized dataset and analysis code supporting the findings of this study are available from the corresponding author upon reasonable request. The data are not publicly deposited because the original informed consent did not explicitly include public sharing of participant-level data.
